# 贝伐珠单抗长期维持治疗晚期非小细胞肺癌39个月的病例报告及相关文献回顾

**DOI:** 10.3779/j.issn.1009-3419.2013.06.10

**Published:** 2013-06-20

**Authors:** 玮 武, 俊舫 唐, 羽华 吴, 允中 朱, 丽艳 徐, 鹤玲 史, 弃逸 孟, 赞 刘, 丽丽 郭, 虹 陶, 明智 李, 喆 刘

**Affiliations:** 101149 北京，首都医科大学附属北京胸科医院肿瘤科 Department of Oncology, Beijing Chest Hospital, Capital Medical University, Beijing 101149, China

**Keywords:** 贝伐珠单抗, 化疗, 肺肿瘤, Bevacizumab, Chemotherapy, Lung neoplasms

## Abstract

本文报道1例女性肺腺癌患者，表皮生长因子受体（epidermal growth factor receptor, *EGFR*）、V-Ki-ras2鼠Kirsten肉瘤病毒致癌基因同源物（V-Ki-ras2 Kirsten rat sarcoma viral oncogene homolog, *KRAS*）基因突变及棘皮动物微管相关类蛋白4与间变性淋巴瘤激酶融合基因（chinodem microtubule-associated protein-like 4/anaplastic lymphoma kinase, *EML4-ALK*）检测结果均为阴性；一线接受贝伐珠单抗（15 mg/kg）联合常规剂量紫杉醇、卡铂方案6周期化疗以及后续贝伐珠单抗的维持治疗。共应用贝伐珠单抗42周期，应用总剂量达44, 730 mg，患者的无进展生存期（progression-free survival, PFS）长达39个月，患者的长期生存获益远超出了不良反应所带来的危害。

## Abstract (en)

We report an advanced stage Chinese female lung adenocarcinoma patient who was negative for epidermal growth factor receptor (EGFR), V-Ki-ras2 Kirsten rat sarcoma viral oncogene homolog (*KRAS*) gene mutations, also negative for chinodem microtubule-associated protein-like 4/anaplastic lymphoma kinase (*EML4-ALK*) gene rearrangement and treated with bevacizumab (15 mg/kg) in combination with 6 cycles of conventional doses of paclitaxel and carboplatin chemotherapy. She was then treated with maintenance bevacizumab for a total of 42 cycles, the total dose of bevacizumab is 44, 730 mg. The progression-free survival was 39 months. Our findings suggest that maintenance bevacizumab for the treatment of non-small cell lung cancer (NSCLC) is safe and its benefit for long-term survival overwhelms its side effects.

## 1 临床资料

患者女性，59岁，无吸烟史，因刺激性干咳6月余于2007年12月17日入院。咳少量白色泡沫痰，无发热、胸痛、胸闷、痰中带血等不适。既往体健，否认高血压、肾脏病等慢性病史。胸部CT检查显示双肺多发团块状阴影，右侧胸膜多发结节影，双侧纵隔淋巴结肿大。经CT定位下肺穿刺活检病理诊断为腺癌，分期T4N3M1。右锁骨上淋巴结活检病理诊断为转移性腺癌，免疫组化结果支持来源于肺。表皮生长因子受体（epidermal growth factor receptor, *EGFR*）、V-Ki-ras2鼠Kirsten肉瘤病毒致癌基因同源物（V-Ki-ras2 Kirsten rat sarcoma viral oncogene homolog, *KRAS*）基因突变检测以及棘皮动物微管相关类蛋白4与间变性淋巴瘤激酶融合基因（chinodem microtubule-associated protein-like 4/anaplastic lymphoma kinase, *EML4-ALK*）融合基因表达结果均为阴性。患者于2008年1月参加了SAiL研究，行6周期紫杉醇（paclitaxel, PTX）、卡铂（carboplatin, CBP）联合贝伐珠单抗（安维汀，Avastin）方案化疗以及化疗后的贝伐珠单抗维持治疗。贝伐珠单抗15 mg/kg d1，PTX 175 mg/m^2^ d1，CBP AUC=6。化疗期间有4级中性粒细胞下降、3级血小板下降、1级鼻出血及齿龈出血、指趾麻木等毒副反应。每2周期复查胸部CT，评价疗效。基线及2、4、6周期化疗后靶病灶长径之和分别为17.0 cm、15.4 cm、11.4 cm及13.1 cm，提示靶病灶与基线相比分别缩小9.4%、32.9%及22.9%，非靶病灶稳定，无新病灶发现，最佳疗效评价为部分缓解（partial response, PR）。6周期化疗后继续应用贝伐珠单抗15 mg/kg，每3周1次，维持治疗至2011年3月30日，在贝伐珠单抗维持治疗期间每6周进行全面复查，评价疗效（图 1A-图 1D）。前后共应用贝伐珠单抗42周期，贝伐珠单抗总剂量为44, 730 mg。于2011年4月19日复查胸部CT，显示靶病灶与最佳疗效相比增大20%，且非靶病灶增多增大，肺内出现新发转移结节，疗效评价进展（progressive disease, PD）（图 1E，图 1F）。患者末次随访时间为2012年9月，PFS为39个月，总生存期（overall survival, OS）为57个月。

**1:**
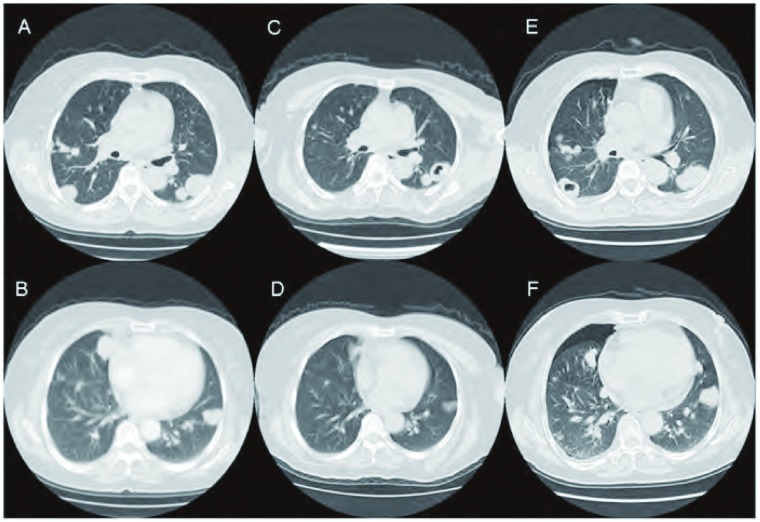
患者治疗前后的胸部CT表现。A、B：基线检查显示双肺多发结节影（2007-12-10）；C、D：6周期贝伐珠单抗合用化疗后显示双肺病变明显缩小及空洞改变（2008-05-19）；E、F：贝伐珠单抗维持治疗39个月时CT显示双肺病变增多增大，病情进展且右肺出现自发性气胸（2011-03-04）。 The computed tomography scans before and after treatment. A, B: The CT scans (10-Dec-2007) of the baseline showed multiple massive and nodular shadows in both lungs; C, D: The CT scans (19-May-2008) after six cycles of chemotherapy and bevacizumab showed that the tumor began shrinking obviously with cavity formation; E, F: The CT scans of the maintenance therapy with bevacizumab for 39 monthes (04-Mar-2011). The tumor sizes had been increasing, pneumothorax in the right lung can be seen and new metastatic nodules were also found in the lungs.

患者在贝伐珠单抗维持治疗期间体力状况评分（performance status, PS）为1分，治疗期间不良反应见表 1，主要副反应为蛋白尿、继发性高血压和自发性气胸等。继发性高血压出现在治疗第6个月，最高达160 Hg/100 mmHg，应用硝苯地平控释片口服治疗后血压控制在正常范围。2011年1月患者曾患轻度自发性气胸，未行特殊治疗，后气胸自行吸收。患者于2008年7月即贝伐珠单抗治疗第6个月开始出现蛋白尿，当时尿常规检查显示尿蛋白（++）。按照方案贝伐珠单抗未减量，继续维持治疗。以后每3周复查24 h尿蛋白定量，尿蛋白定量 < 2.0 g/kg时继续应用，否则暂停贝伐珠单抗治疗，每3周复查，至24 h尿蛋白定量符合应用标准时继续应用。患者从贝伐珠单抗治疗第19个月开始，尿蛋白开始出现 > 2 g/24 h，故予以第1次暂停贝伐珠单抗。自2009年8月至治疗结束共发生因尿蛋白副反应暂停贝伐珠单抗应用7次，其中最长一次停药时间持续约3个月。24 h尿蛋白定量的最高值为3, 926 mg/24 h，按照国立癌症研究所常规毒性评价标准（National Cancer Institute-Common Terminology Criteria, NCI-CTC）分级3级，出现在贝伐珠单抗治疗的第33个月。采用中西医结合治疗蛋白尿，先后曾应用贝那普利及口服百令胶囊等中药治疗，结果显示贝伐单抗的暂时停用并结合口服中药治疗可使尿蛋白定量数值出现下降。

**1:** 贝伐珠单抗治疗期间的主要不良反应 Adverse events occurred during maintenance therapy with bevacizumab

Adverse event	Occurrence time^*^ (month)	Grade	Recovery time^*^ (month)
Gum bleeding	1	1	12
spontaneous pneumothorax	36	1	43
Hypertension	6	2	41
Proteinuria	6	3	Proteinuria(+)^**^
^*^from the first bavacizumab infusion. ^**^one year after stop use of bavacizumab treatment.			

## 2 讨论

贝伐珠单抗联合化疗目前成为非鳞型非小细胞肺癌（non-small cell lung cancer, NSCLC）患者标准的一线治疗方案。许多肿瘤治疗指南推荐将化疗联合贝伐珠单抗作为晚期NSCLC的一线治疗方案^[1, 2]^。贝伐珠单抗作用于血管内皮生长因子（vascular endothelial growth factor, VEGF）通路，可通过精确抑制VEGF，使肿瘤新生血管退化，存活血管正常化及抑制血管再生，从而持续抑制肿瘤生长和转移^[3-5]^。

2006年发表在《新英格兰医学杂志》上的ECOG4599研究^[6]^采用贝伐珠单抗联合化疗一线治疗晚期非鳞NSCLC，结果显示：与紫杉醇+卡铂联合安慰剂相比，紫杉醇+卡铂联合贝伐珠单抗方案改善了患者的有效率、PFS和OS，化疗联合贝伐珠单抗方案可使中位OS延长2个月。2年后的AVAiL研究^[7]^从有效率和中位无进展生存期方面证实了ECOG4599的结果。

近期发表了两篇荟萃分析^[8, 9]^，对所有已发表的比较化疗联合贝伐珠单抗与单纯化疗方案一线治疗局部晚期或转移性NSCLC疗效的随机对照研究进行系统评价和分析，结果均在不同程度上证实了贝伐珠单抗的治疗益处。

在目前NSCLC的维持治疗中，抗肿瘤血管生成治疗已经成为维持治疗的模式之一。多项大型临床研究证实在一线含铂化疗结束后，继续应用贝伐珠单抗维持治疗可使患者获得最长的生存获益。我们回顾总结了近期含贝伐珠单抗治疗临床试验的结果（表 2），其中应用贝伐治疗最长的是在SAiL研究中，时间为132.3周。

**2:** 贝伐珠单抗治疗晚期非小细胞肺癌的主要临床试验 Clinical trials combining bevacizumab in the treatment of advanced non-small cell lung cancer

Study	Patient enrolled	Treatment regimen	Median PFS (month)	Median OS (month)	Median cycles of Bev	Median duration of Bev (week)
E4599: Sandler *et al*.^[6]^	878	CP-Bev	6.2	12.3	7	NA
AVAIL: Reck *et al*.^[7]^	1, 043	CG-Bev 7.5 mg	6.8	13.6	6 (1- > 18)	19.6
		CG-Bev 15 mg	6.6	13.4	5 (1- > 18)	17.6
SAiL: Crinò *et al*.^[10]^	2, 212	Carboplatin doublets-Bev	7.6 (7.2-8.1)	14.3 (13.2-15.6)	7 (1-43)	21.3 (0.1-132.3)
JO19907: Niho *et al*.^[11]^	180	CP-Bev	6.9 (6.1-8.3)	22.8 (17.4-28.5)	NA	NA
AVAPERL: Barlesi *et al*.^[12]^	376	CPem-Bev Bev maintenance	6.6	15.7	NA	NA
		CPem-Bev Pem+Bev maintenance	10.2	> 15.7	NA	NA
ABIGAIL: Mok *et al*.^[13]^	303	CG or CP Bev 7.5 mg	6.8	13.4	NA	NA
		CG or CP Bev 15 mg	6.7	13.7	NA	NA
AVF0705: Johnson *et al*.^[14]^	99	CP-Bev 7.5 mg	4.3 (0.2-12.9)	11.6 (0.2-56.8)	10 (1-18)	12 (0.2- > 24)
		CP-Bev 15 mg	7.4 (0.7-12.5)	17.7 (0.8-57.8)		
Patel *et al*. ^[15]^	50	CP-Bev	7.8 (5.2-11.5)	14.1 (10.6-19.6)	7 (1-51)	NA
BRIDGE: Hainsworth *et al*.^[16]^	31	CP-Bev	6.2 (5.32-7.62)	NA	6 (1-18)	NA
CP: Carboplatin/Paclitaxel; CG: Cisplatin/Gemcitabine; Bev: Bevacizumab; CPem: Cisplatin/Pemetrexed; NA: not applicable.						

2010年8月发表了大型Ⅳ期临床研究SAiL的结果^[10]^，包括中国在内的2, 212例患者参加了该项研究，其中1, 332例患者接受了贝伐珠单抗的维持治疗。结果显示，中位疾病进展时间（time to progression, TTP）为7.8个月，中位OS为14.6个月。其中亚洲患者314例，TTP为8.3个月，中位OS为18.9个月，明显高于总体人群。SAiL研究进一步验证了贝伐珠单抗联合化疗一线治疗非鳞NSCLC的生存优势。

本例患者治疗按照标准的紫杉醇与卡铂联合方案化疗及贝伐珠单抗15 mg/kg治疗6周期。化疗结束后继续应用贝伐珠单抗维持治疗至疾病进展。该患者共应用贝伐珠单抗42周期，PFS达39个月，取得了较长的生存期；经检索，这是首例肺癌患者接受贝伐珠单抗治疗（含维持）超过36个月的病例报道，该病例也说明了对于化疗联合贝伐珠单抗一线治疗后疾病缓解及稳定的患者，可能会受益于贝伐珠单抗的维持治疗。

本例患者在贝伐珠单抗治疗过程中出现的主要不良反应为继发性高血压和无症状蛋白尿。高血压是常见的贝伐珠单抗治疗相关不良反应，发生率为8%-67%，其中多数患者为3级以下的轻中度血压升高，3级以上的严重高血压发生率为5%-18%^[17]^。本文患者应用贝伐珠单抗治疗共39个月，其中单药维持治疗35个月。在贝伐珠单抗治疗第6个月开始出现了NCI-CTC分级2级的继发性高血压，应用一种抗高血压药物治疗，血压可控制在正常范围，血压增高程度未随贝伐珠单抗的应用时间及累计剂量增加而加重。

蛋白尿是另一类常见的贝伐珠单抗治疗相关不良反应，发生率约为0.7%-38%，大部分为无症状蛋白尿，3级蛋白尿的发生率 < 3%，4级蛋白尿的发生率 < 1%^[17]^。荟萃分析^[18]^显示蛋白尿的发生与贝伐珠单抗呈剂量依赖性。本文患者在贝伐珠单抗治疗第6个月开始出现无症状蛋白尿，初始蛋白尿CTC分级为1级。贝伐珠单抗治疗第14个月时首次出现CTC分级2级蛋白尿。24 h尿蛋白定量的最高值3, 926 mg/24 h（CTC分级3级）出现在贝伐珠单抗治疗的第33个月。贝伐珠单抗维持治疗期间的尿蛋白呈现随药物应用时间及累计剂量增加的进行性上升趋势。治疗过程中遵循24 h尿蛋白 > 2 g即暂停贝伐珠单抗治疗的原则。治疗经验显示贝伐珠单抗的暂时停用并结合口服中药治疗可使尿蛋白定量数值出现下降。值得关注的是在患者停用贝伐珠单抗1年后的尿常规检查仍显示尿蛋白为1+。

有文献^[19]^报道在应用贝伐珠单抗治疗肺癌的过程中可能会出现自发性气胸，可能的原因主要为病变位于肺外周，甚至已经侵犯了胸膜，损伤胸膜形成支气管胸膜瘘；其次，与肿瘤近远端气道的单向阀原理及肿瘤栓子引起的肺梗塞可能形成气体渗漏等因素有关。

综上所述，本例患者应用贝伐珠单抗15 mg/kg治疗39个月，与贝伐珠单抗长期使用的相关主要不良反应为继发性高血压、蛋白尿及自发性气胸。患者的治疗相关不良反应可以耐受，未影响生存质量，反映出该患者长期应用贝伐珠单抗维持治疗的安全性良好，患者得到的长期生存获益远远超出了不良反应所带来的危害。
